# *Arabidopsis* Transcription Factor TCP5 Controls Plant Thermomorphogenesis by Positively Regulating PIF4 Activity

**DOI:** 10.1016/j.isci.2019.04.005

**Published:** 2019-05-08

**Authors:** Xiang Han, Hao Yu, Rongrong Yuan, Yan Yang, Fengying An, Genji Qin

**Affiliations:** 1 State Key Laboratory of Protein and Plant Gene Research, School of Life Sciences, Peking University, Beijing 100871, The People's Republic of China; 2 School of Advanced Agricultural Sciences, Peking University, Beijing 100871, People's Republic of China; 3 The Peking-Tsinghua Center for Life Sciences, Beijing 100871, The People's Republic of China; 4 Academy for Advanced Interdisciplinary Studies, Peking University, Beijing 100871, The People's Republic of China; 5 Academy for Advanced Interdisciplinary Studies, Southern University of Science and Technology (SUSTech), Shenzhen 518055, China

**Keywords:** Biological Sciences, Molecular Biology, Plant Biology

## Abstract

Plants display thermomorphogenesis in response to high temperature (HT). PHYTOCHROME INTERACTING FACTOR 4 (PIF4) is a central integrator regulated by numerous negative regulators. However, the mechanisms underpinning PIF4 positive regulation are largely unknown. Here, we find that TEOSINTE BRANCHED 1/CYCLOIDEA/PCF 5 (TCP5), TCP13, and TCP17 transcription factors promote the activity of PIF4 at transcriptional and post-transcriptional levels. *TCP5* is rapidly induced by HT treatment, and TCP5 protein stability increases under HT. The overexpression of *TCP5* causes constitutive thermomorphogenic phenotypes, whereas the *tcp5 tcp13 tcp17* triple mutant exhibits aberrant thermomorphogenesis. We demonstrate that TCP5 not only physically interacts with PIF4 to enhance its activity but also directly binds to the promoter of *PIF4* to increase its transcript. TCP5 and PIF4 share common downstream targets. The *tcp5 tcp13 tcp17* mutant partially restores the long hypocotyls caused by *PIF4* overexpression. Our findings provide a layer of understanding about the fine-scale regulation of PIF4 and plant thermomorphogenesis.

## Highlights

• TCP5, TCP13, and TCP17 positively regulate plant thermomorphogenesis
• TCP5 directly interacts with PIF4 transcription factor
• TCPs and PIF4 share common downstream target genes
• TCP5 promotes PIF4 activity at both transcriptional and protein levels

## Graphical Abstract

**Figure:**
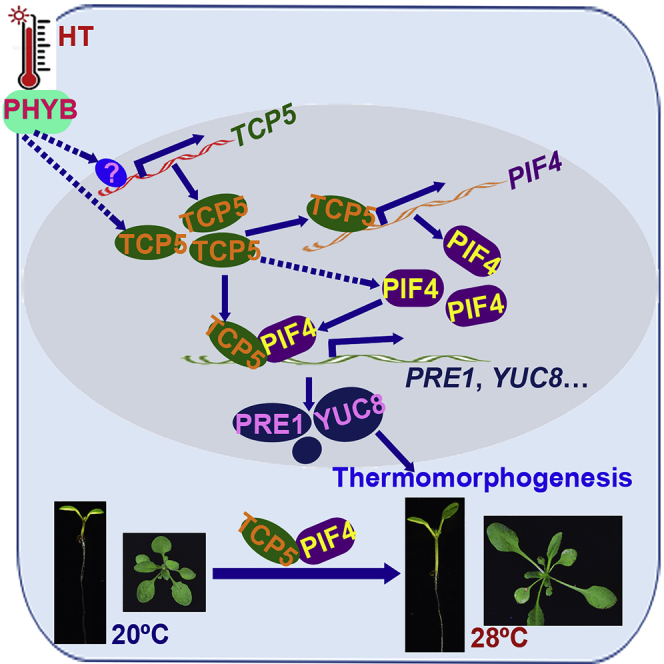

## Introduction

The strong 2018 summer heat waves across the Northern Hemisphere caused a high temperature (HT) of up to 30°C in some areas of the Arctic Circle and also severe losses in crop production. There is an urgent need to elucidate the molecular mechanisms by which plants adapt to HT. Plant thermomorphogenesis refers to changes in plant growth, development, and morphology under HT (Stavang et al., 2009). These changes, including hypocotyl elongation, petiole elongation, and hyponastic growth, are important for plant survival under HT (Quint et al., 2016, Wigge, 2013). The basic-helix-loop-helix (bHLH) transcription factor PHYTOCHROME INTERACTING FACTOR 4 (PIF4) integrates HT and other environmental cues with hormonal signals and acts as a central hub in the control of thermomorphogenesis (Franklin et al., 2011, Koini et al., 2009, Sun et al., 2012), and its activity is tightly regulated by numerous negative regulators (Bernardo-Garcia et al., 2014, Box et al., 2015, Delker et al., 2014, Foreman et al., 2011, Gangappa and Kumar, 2017, Jung et al., 2016, Legris et al., 2016, Ma et al., 2016, Nieto et al., 2015, Nusinow et al., 2011, Somers et al., 1991, Song et al., 2017, Tasset et al., 2018, Toledo-Ortiz et al., 2014, Zhang et al., 2017, Zhang et al., 2018). At the transcriptional level, *PIF4* transcripts are negatively regulated by the light-signaling component LONG HYPOCOTYL 5 (HY5) (Gangappa and Kumar, 2017, Toledo-Ortiz et al., 2014) and the evening complex component EARLY FLOWERING 3 (ELF3) (Nieto et al., 2015, Nusinow et al., 2011). At the protein level, the phosphorylation of PIF4, which is required for its degradation, is mediated by the BRASSINOSTEROID-INSENSITIVE 2 (BIN2) kinase in brassinosteroid (BR) signaling and by phytochrome B (phyB), which acts as both a photoreceptor and a thermosensor (Bernardo-Garcia et al., 2014, Jung et al., 2016, Legris et al., 2016, Song et al., 2017). Recently, BLADE-ON-PETIOLE (BOP) proteins, a component of CUL3^BOP1/BOP2^ (CULLIN3A^BOP1/BOP2^) E3 ubiquitin ligase complex, have been reported to mediate PIF4 degradation (Zhang et al., 2017). Several proteins, such as CRYPTOCHROME 1 (CRY1) (Ma et al., 2016), LONG HYPOCOTYL IN FAR-RED 1 (HFR1) (Foreman et al., 2011), and ELF3 (Box et al., 2015), repress PIF4 transcriptional activity by directly interacting with PIF4. However, to date, apart from BRASSINAZOLE RESISTANT 1 (BZR1) (Ibanez et al., 2018, Oh et al., 2012, Oh et al., 2014), very few PIF4 positive regulators have been found. Our previous work has found that *Arabidopsis* transcriptional repressor SPOROCYTELESS/NOZZLE (SPL/NZZ) inhibits the activity of CINCINNATA (CIN)-like TCP family and that the overexpression of *TCP5* leads to aborted ovules (Wei et al., 2015). TCP proteins are a conserved, plant-specific class of transcription factors (Martín-Trillo and Cubas, 2010). They are further grouped into Class I and Class II based on their conserved TCP domains, which are responsible for DNA binding or protein-protein interactions (Martín-Trillo and Cubas, 2010). TCPs play essential roles in the control of plant development (Aggarwal et al., 2010, Aguilar-Martinez et al., 2007, Efroni et al., 2008, Gonzalez-Grandio et al., 2013, Kieffer et al., 2011, Koyama et al., 2007, Koyama et al., 2010, Nath et al., 2003, Palatnik et al., 2003, Yang et al., 2018), such as internode length (Kieffer et al., 2011), leaf shape (Efroni et al., 2008, Koyama et al., 2007, Koyama et al., 2010), and axillary branching (Aguilar-Martinez et al., 2007, Gonzalez-Grandio et al., 2013). However, TCPs have not been found to be in participating in the regulation of plant thermomorphogenesis yet. Here, we show that TCP5, TCP13, and TCP17 act as positive regulators in plant response to HT. Overexpression of *TCP5*, *TCP13,* or *TCP17* leads to constitutive thermomorphogenesis, whereas the triple mutant *tcp5tcp13tcp17* displays aberrant thermomorphogenesis. TCP5 is accumulated under HT and directly interacts with the central regulator PIF4. We finally demonstrate that TCP5 plays an essential role in plant thermomorphogenesis by promoting the activity of PIF4 at both transcriptional and post-transcriptional levels.

## Results and Discussion

### TCP5 Positively Regulates Plant Thermomorphogenesis

**Figure 1:**
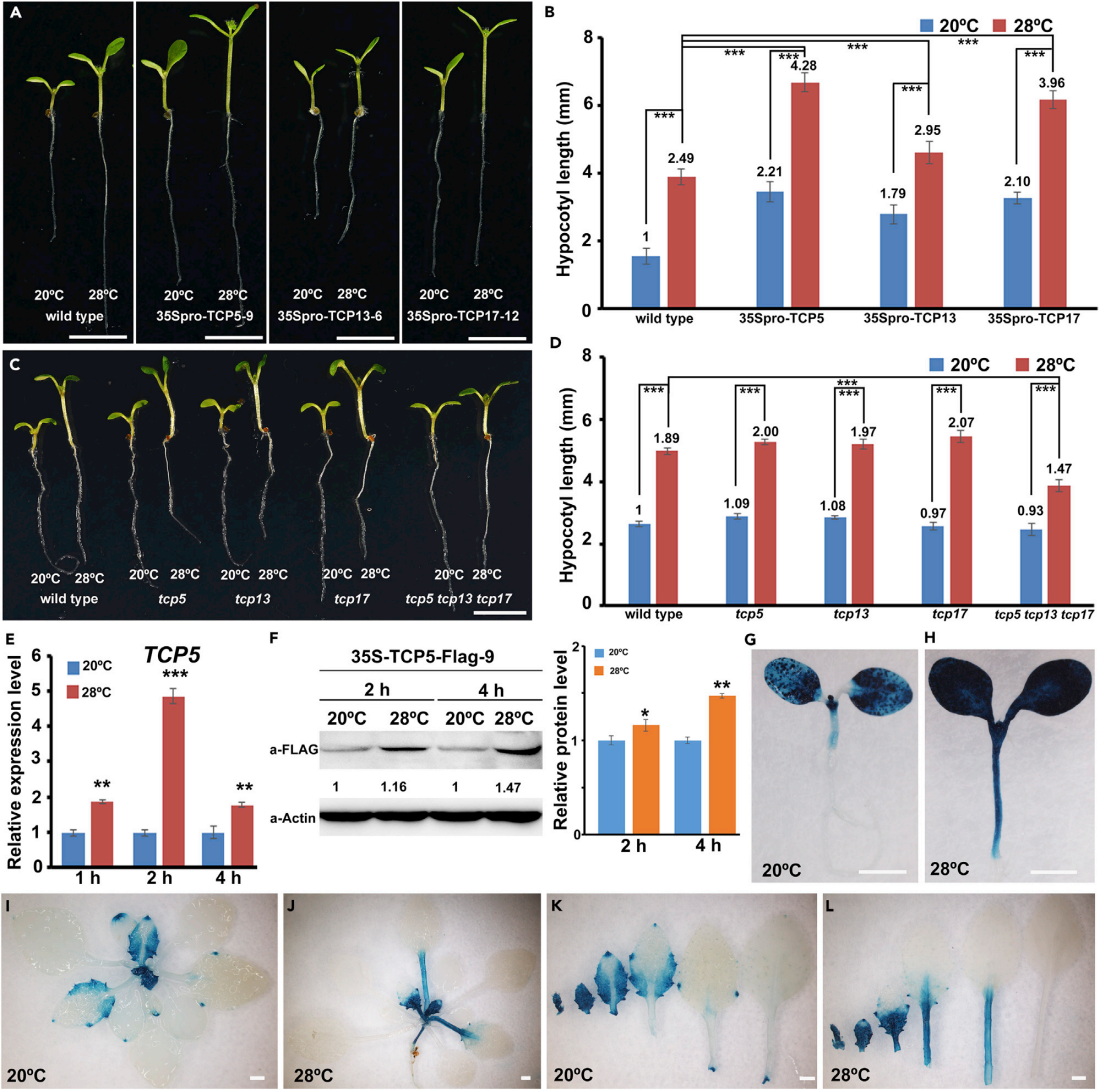
TCP5 Regulates Plant Thermomorphogenesis (A) Phenotypes of 10-day-old wild-type, 35Spro-TCP5-9, 35Spro-TCP13-6, and 35Spro-TCP17-12 seedlings grown continuously at 20°C for 7 days before being transferred to 28°C for 3 days. (B) Statistical analysis of the hypocotyl lengths of the plants in (A). (C) Phenotypes of 10-day-old wild-type, *tcp5*, *tcp13*, *tcp17,* and *tcp5 tcp13 tcp17* triple mutant seedlings after HT treatment. (D) Statistical analysis of the hypocotyl lengths of the plants in (C). (B and D) Data represent the mean ± SD of three biological replicates. Significant differences are indicated with *p < 0.05, n > 20, two-tailed Student's t test; **p < 0.01; ***p < 0.001. The number above each column indicates fold changes of hypocotyl length relative to wild-type control under 20°C. (E) Relative expression levels of *TCP5* in wild-type after HT treatment for 1, 2, or 4 h. The expression levels of *TCP5* were normalized to that of *ACT8* and were relative to that in wild-type grown under normal temperatures. Data represent the mean ± SD of three biological replicates. (F) Stability of TCP5 protein under HT treatment. 35Spro-TCP5-FLAG plants were treated with HT for 2 and 4 h. (G and H) GUS staining of 10-day-old TCP5pro-GUS transgenic seedlings after treatment. Seven-day-old TCP5pro-GUS seedlings grown under continuous 20°C before being transferred to 20°C (G) or 28°C (H) for 3 additional days of growth. (I) GUS staining of 26-day-old TCP5pro-GUS plants grown under normal temperature. (J) GUS staining of 26-day-old TCP5pro-GUS plants treated with HT for 5 days. (K) Close-up view of dissected leaves from (I). (L) Close-up view of dissected leaves from (J). Scale bars, 5 mm in (A and C) and 1 mm in (G–L). See also Figure S1.

We previously found that the overexpression of *TCP5* leads to aborted ovules (Wei et al., 2015). Surprisingly, we observed that most 35Spro-TCP5 overexpression lines (32/41) displayed constitutive thermomorphogenesis, including long hypocotyls, long petioles, and increased leaf hyponasty under normal temperatures (Figures 1A, 1B, and S1A–S1E), implying that TCP5 might play important roles in plant thermomorphogenesis. The protein alignments suggest that TCP5 is highly similar to TCP13 and TCP17, and that they form a small clade in the Class II TCP family (Figures S1F and S1G). To test whether TCP13 and TCP17 could be functionally redundant with TCP5, we overexpressed *TCP13* or *TCP17* using the Cauliflower Mosaic Virus (CaMV) 35S promoter and found that both 35Spro-TCP13 and 35Spro-TCP17 transgenic plants exhibited longer hypocotyls at 20°C and 28°C, resembling the constitutive thermomorphogenesis observed in 35Spro-TCP5 plants (Figures 1A and 1B). We then investigated the hypocotyl lengths of *tcp5*, *tcp13*, and *tcp17* single mutants and of a *tcp5tcp13tcp17* triple mutant under 28°C treatment for 3 days. The single mutants displayed no differences from the wild-type in hypocotyl length at 20°C or 28°C, whereas the triple mutant produced shorter hypocotyls than the wild-type under 28°C (Figures 1C and 1D). We further found that the petiole length of *tcp5 tcp13 tcp17* was shorter than that of the wild-type under 28°C (Figures S1H and S1I), indicating that *tcp5 tcp13 tcp17* mutants were insensitive to HT treatment. These results demonstrate that TCP5 has functional redundancy with TCP13 and TCP17 in the positive regulation of plant thermomorphogenesis.

### HT Regulates TCP5 at Transcriptional and Post-transcriptional Levels

To test whether the expression level of *TCP5*, *TCP13,* and *TCP17* could be regulated by HT, we first treated wild-type *Arabidopsis* seedlings with HT in a time course (1, 2, or 4 h). Quantitative real-time PCR (qRT-PCR) assays showed that the transcripts of *TCP5*, *TCP13,* and *TCP17* were all rapidly induced by HT (Figures 1E, S1J, and S1K). PIF4 is a central regulator in plant response to HT (Franklin et al., 2011, Koini et al., 2009, Sun et al., 2012). We then tested the expression levels of *TCP5* in *pif4* mutant with or without HT treatment. The results showed that the induction of *TCP5* in HT was significantly reduced (Figure S1L). We further investigated TCP5 protein stability using 35Spro-TCP5-FLAG transgenic plants. The results indicated that TCP5 was more stable under HT than under normal conditions (Figure 1F). To investigate the expression pattern of *TCP5*, we generated a TCP5pro-GUS construct in which the *GUS* reporter gene was driven by the 2,400-bp *TCP5* promoter. Fifteen TCP5pro-GUS transgenic lines showed similar GUS staining patterns, and strong GUS activity was observed in the cotyledons, hypocotyls, young leaf blades, and petioles (Figures 1G, 1I, 1K, and S1M–S1O). As the leaves grew, GUS staining gradually faded from the petioles and from the middle region of the blades to the leaf margins, which were mainly restricted to the leaf margins or serrations (Figures 1I, 1K, S1N, and S1O). GUS staining was also observed in the petals, anthers, pistils, and siliques (Figures S1P and S1Q). In addition, we also detected GUS signals in ovule and pollen grains in TCP5pro-GUS transgenic plants (Figures S1R and S1S). Furthermore, 28°C HT treatment for 3 days strengthened the GUS activity in the hypocotyls and cotyledons of TCP5pro-GUS-4 plants (Figures 1G and 1H). Interestingly, HT treatment for 3 days led to strong GUS activity shifting from the leaf blades to the petioles (Figures 1I–1L), consistent with the petiole elongation and reduced blade areas under HT. These observations further indicate that TCP5 acts as an important factor in the control of plant responses to HT.

### TCP5 Acts as a Transcriptional Activator

To determine the subcellular localization of TCP5, we generated TCP5pro-TCP5-GFP in which the *TCP5* promoter was used to drive the *TCP5-GFP* fusion gene. We observed clear green fluorescence in the nuclei of hypocotyl cells of TCP5pro-TCP5-GFP transgenic plants, consistent with the function of TCP5 as a transcription factor (Figures S2A–S2D). We then used a GAL4-UAS-LUC reporter system (Guo et al., 2015, Tao et al., 2013) to test transactivation activity of TCPs. When the reporter was co-transformed with either GAL4DBD-PIF4 or GAL4DBD-TCP5, constructs in which the CaMV 35S promoter drives *PIF4* or *TCP5* fused to the sequence encoding the GAL4 DNA-binding domain (DBD), the *LUC* reporter gene was clearly activated, whereas in the negative controls, no fluorescence signal was observed (Figure S2E), indicating that TCP5 and PIF4 were transactivators. We further showed that the TCP domain was required for the transactivation activity of TCP5 because a truncated TCP5 with the TCP domain deleted could not activate the expression of reporter gene (Figure S2F). We also demonstrated that TCP13 and TCP17 were transactivators and that the TCP domain was required for their transactivation activity (Figures S2G and S2H).

### TCP5 Interacts with PIF4

**Figure 2:**
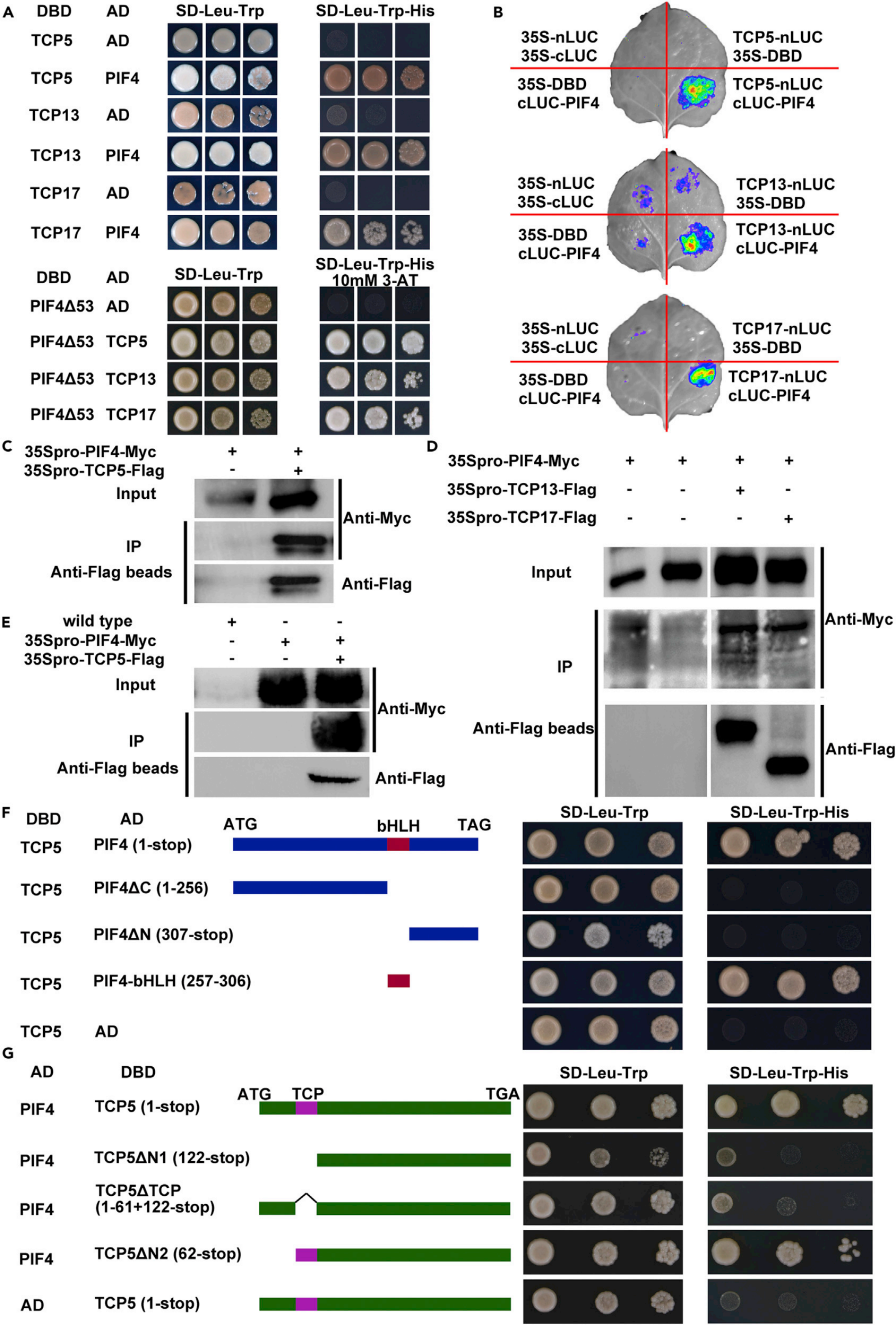
TCP5 Interacts with PIF4 (A) Yeast two-hybrid assays showing that TCP5, TCP13, and TCP17 interact with PIF4. The DNA-binding domain (DBD) was fused to TCPs or truncated PIF4Δ53, in which 53 amino acids at the N-terminal end were deleted. The activation domain (AD) was fused to PIF4 or TCPs. Co-transformed yeast cells were grown on medium lacking Leu and Trp (SD-Leu-Trp) and selected on medium lacking Leu, Trp, and His (SD-Leu-Trp-His) with or without 10 mM 3-amino-1,2,4 triazole (3-AT) at dilutions of 10- and 100-fold. The empty vector pDEST22 was used as a negative control. (B) Firefly luciferase complementation assays in tobacco leaves confirmed the interactions between TCPs and PIF4. (C) Co-immunoprecipitation (coIP) indicated that PIF4 could be combined with TCP5 when both were transiently expressed in tobacco leaves. (D) CoIP suggested that TCP13 and TCP17 could interact with PIF4 in tobacco leaves. (E) CoIP with 35Spro-TCP5-FLAG 35Spro-PIF4-Myc transgenic plants confirmed the interaction between TCP5 and PIF4. Wild-type plants were used as negative controls. (F) Analysis of PIF4 truncations suggested that the bHLH domain of PIF4 was responsible for the interaction with TCP5. (G) Analysis of TCP5 truncations suggested that the TCP domain of TCP5 was required for the interaction with PIF4. See also Figure S2.

To reveal the molecular mechanisms by which TCP5 controls plant thermomorphogenesis, we performed a yeast two-hybrid screen (Ou et al., 2011) using TCP5 as bait proteins and finally identified that PIF4 interacted with TCP5. We further confirmed this interaction by yeast two-hybrid assays, and results showed that TCP5, TCP13, and TCP17 all interacted with PIF4 fused with either the GAL4 activation domain or DBD (Figure 2A). Firefly luciferase complementation imaging indicated that TCP5, TCP13, and TCP17 indeed interacted with PIF4 (Figure 2B). We then confirmed the interaction of PIF4 with TCP5, TCP13, and TCP17 via co-immunoprecipitation (coIP) assays after transient expression in tobacco leaves (Figures 2C and 2D). We further used transgenic plants expressing PIF4-Myc and TCP5-FlAG to conduct coIP assays in planta, and the results indicated that TCP5 indeed interacted with PIF4 (Figure 2E). To identify the domains in TCP5 and PIF4 responsible for their interaction, we first deleted the C or N terminus, which includes the bHLH domain, of PIF4. The PIF4 deletions without the bHLH domain did not interact with TCP5 (Figure 2F). In addition, the bHLH domain of PIF4 was necessary and sufficient for the interaction between PIF4 and TCP5 (Figure 2F). We then made deletions of TCP5, and the results suggested that the TCP domain of TCP5 was required for its interaction with PIF4 (Figure 2G). These data suggested that TCP5 interacted with PIF4 and that their DBDs were necessary for TCP5-PIF4 interaction. To test whether TCP5 could also interact with PIF4 homologs including PIF1, PIF3, PIF5, and PIF7, which are key regulators in light signaling (Leivar et al., 2008, Leivar and Quail, 2011), we carried out the yeast two-hybrid assays. The results showed that PIF1, PIF3, PIF5, and PIF7 all interacted with TCP5 (Figure S2I), suggesting that TCP5 might be also important for plant photomorphogenesis.

### TCP5 and PIF4 Regulates Common HT-Induced Genes

To identify the downstream targets of TCP5 and provide more evidence supporting the association of TCP5 with PIF4, we conducted RNA sequencing (RNA-seq) transcriptome analysis using petioles or blades from wild-type grown at normal temperatures (20°C), and wild-type, *tcp5tcp13tcp17* mutants, and *pif4* mutants treated with HT (28°C) for 6 h. We identified 1,081 differentially expressed genes (DEGs) (false discovery rate < 0.01; fold change ≥ 2.0 or ≤ -2.0) (430 HT-induced genes and 651 HT-repressed genes) in petioles (Table S1) after HT treatment. By contrast, only 558 DEGs (240 HT-induced genes and 318 HT-repressed genes) were found in the blades (Table S1), suggesting that the genes in the blade were less affected than those of the petiole by HT treatment. Furthermore, less than 13% (76/594) of HT-induced genes and approximately 20% (163/806) of HT-repressed genes were shared between the petioles and blades (Figures S3A and S3B). Interestingly, heatmaps of 594 HT-induced genes and 806 HT-repressed genes in leaves showed that the difference in the expression pattern of most genes in petioles was opposite in blades (Figures S3C and S3D). It is known that sessile plants display differential organ growth for adaptation in response to environmental cues. For example, light has opposite effects on the growth of cotyledons and hypocotyls. HT even differentially regulates the growth of two parts of one organ by promoting leaf petiole elongation and inhibiting the expansion of leaf blades. Recently, it has been shown that light differentially controls the expression of a class of *Small Auxin Up RNA* (*SAUR*) genes, which are responsible for the differential growth of cotyledons and hypocotyls under light (Sun et al., 2016). Our transcriptome data revealed that a large amount genes were differentially expressed in leaf blades and leaf petioles after HT treatment (Figures S3C and S3D). These genes might also be very important for the opposite effects of HT on the growth of the two parts of leaves under HT.

**Figure 3:**
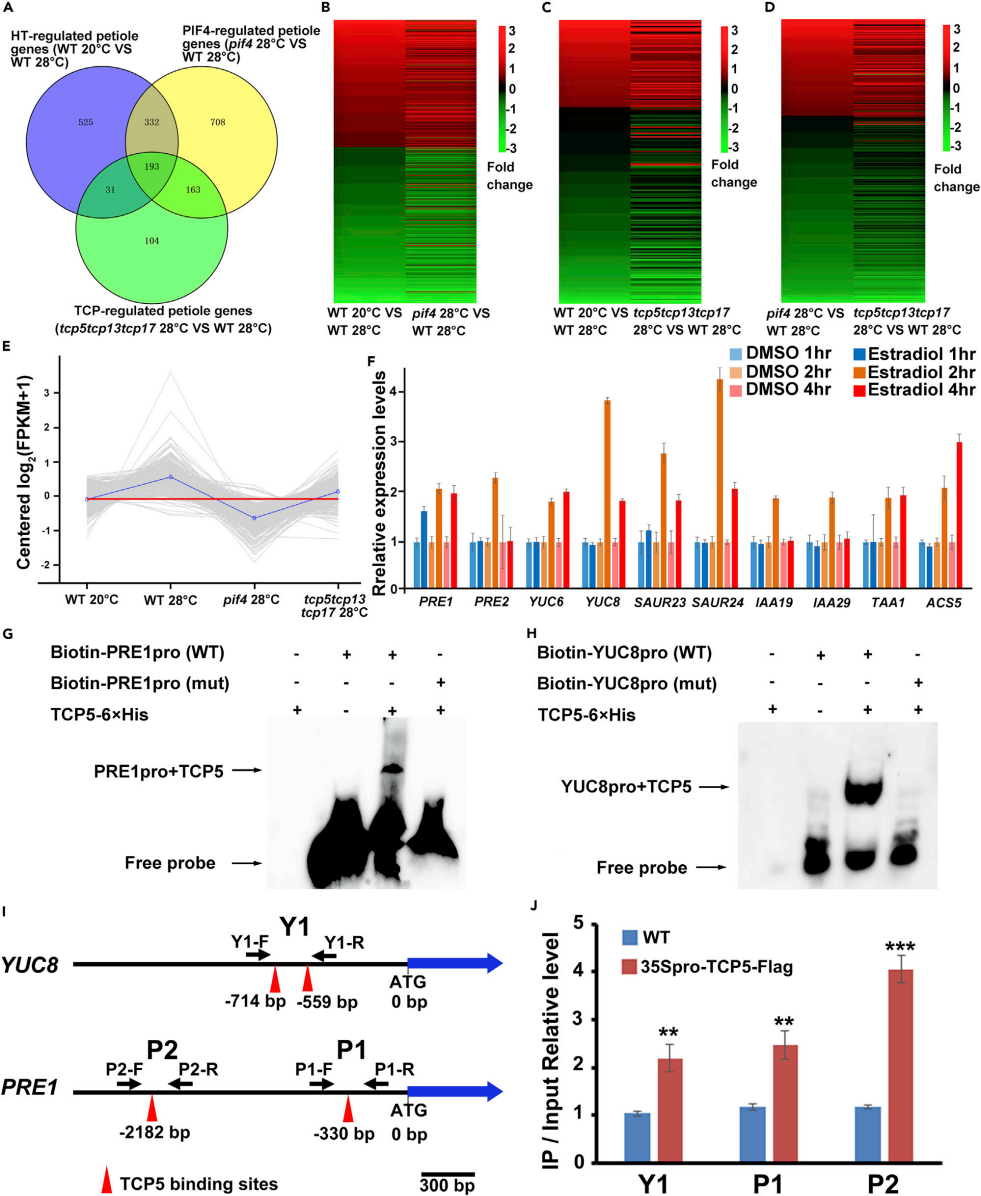
TCP5 and PIF4 Co-regulate Common Target Genes (A) The Venn diagram indicates the numbers of differentially expressed genes (DEGs) coregulated by high temperature (HT), PIF4, and TCPs. (B) Heatmap of 525 DEGs coregulated by HT and PIF4. (C) Heatmap of 224 DEGs coregulated by HT and TCPs. (D) Heatmap of 356 DEGs coregulated by PIF4 and TCPs. (E) Trend lines of 208 DEGs upregulated by HT and PIF4 suggested that most of these genes were also regulated by TCPs. The gray lines represent the relative expression of each gene. The blue line represents the mean relative expression of all genes. (F) qRT-PCR analysis of known PIF4 direct target genes in TCP5-inducible pER8-iTCP5 transgenic plants. The pER8-iTCP5 transgenic plants were treated with 50 μM estradiol or DMSO for 1, 2 or 4 h. The gene expression levels were normalized to that of *ACT8* and are relative to that in pER8-iTCP5 treated with DMSO. The data represent the mean ± SD of three biological replicates. (G) EMSA showed that TCP5 directly binds the promoter of *PRE1*. (H) EMSA indicated that TCP5 directly binds the promoter of *YUC8*. (I) Diagrams showing TCP5 binding motif in the promoter region of *YUC8* and *PRE1*. Y1-F/Y1-R, P1-F/P1-R, and P2-F/P2-R indicate PCR primer pairs used in (J). (J) ChIP-PCR assays showed that TCP5 bound to the promoter region of *YUC8* and *PRE1*. 14-day-old wild-type (WT) and 35Spro-TCP5-FLAG plants grown under 20°C condition were collected for ChIP-PCR using the primer pairs as shown in (I). The data were first normalized to *ACT8* and then were relative to that of input DNA samples. The data represent mean ± SD of three biological replicates. See also Figure S3, Table S1, S2, and S3.

As the transcriptomic changes in petioles were more obvious than those in blades, we then focused on comparing the petiole transcriptomes of *tcp5 tcp13 tcp17* or *pif4* mutants with those of wild-type under HT (28°C). Among the 1,396 PIF4-regulated petiole genes (DEGs between *pif4* mutants and wild-type under 28°C) (Table S2), more than 37% (525/1,396) of the genes were shared with HT-regulated genes (DEGs from wild-type with HT treatment) (Figure 3A), whereas almost 45% (224/491) of TCP-regulated petiole genes (DEGs between *tcp5 tcp13 tcp17* mutant and wild-type under 28°C) (Table S2) were included in HT-regulated genes (Figure 3A), suggesting that both PIF4 and TCPs played important roles in thermomorphogenesis. Interestingly, approximately 72% (356/491) of TCP-regulated genes were also regulated by PIF4 in petioles (Figure 3A). Gene Ontology analysis of these genes regulated by both TCPs and PIF4 showed that the pathway genes responding to abiotic or external and hormonal or internal stimuli were significantly enriched in the upregulated genes, whereas the cold pathway genes were enriched in the downregulated genes (Figures S3E and S3F), consistent with the important roles of PIF4 and TCPs in plant response to HT. Moreover, heatmap analysis showed that most of these coregulated genes were regulated in the same direction by HT and PIF4 (Figure 3B), HT and TCP (Figure 3C), or TCP and PIF4 (Figure 3D). These data provide strong evidence for the association of TCPs with PIF4 during thermomorphogenesis.

### TCP5 and PIF4 Shares Direct Target Genes including *PRE1* and *YUC8*

To find the direct target genes of TCP5, we first analyzed whether the downstream genes of PIF4 and HT could also be regulated by TCPs. Among 525 genes coregulated by HT and PIF4, 208 genes were upregulated. We retrieved the expression data for these 208 genes in petioles from RNA-seq data (Table S3). H-cluster (hierarchical cluster) analysis showed that the expression levels of most genes in *tcp5 tcp13 tcp17* mutants under HT were lower than those in wild-type under HT but higher than those in *pif4* mutants under HT (Figure 3E), implying that TCPs could enhance PIF4 activity. These 208 genes include many PIF4 direct target genes, such as *PRE1* (Bai et al., 2012, Oh et al., 2012), *IAA19* (Sun et al., 2013), and *YUC8* (Sun et al., 2012) (Figure S3G). We further used qRT-PCR to test the changes in the expression of known PIF4 direct target genes in wild-type, *pif4* mutant, and *tcp5 tcp13 tcp17* mutant after HT treatment. The results of qRT-PCR showed that the expression levels of *YUC8*, *PRE1,* and *IAA19* were rapidly upregulated in the petioles and hypocotyls of wild-type plants but were not or less induced in those of *pif4* mutant and *tcp5 tcp13 tcp17* mutant after HT treatment (Figures S3H and S3I). Moreover, we generated pER8-iTCP5 estrogen-inducible transgenic plants, and we found that pER8-iTCP5 plants produced longer hypocotyls after estrogen treatment (Figures S3J and S3K). We then briefly treated pER8-iTCP5 with estrogen in a time course (1, 2, or 4 h) to clarify downstream targets of TCP5. The qRT-PCR results showed that PIF4 direct target genes, including *PRE1* and *YUC8*, were rapidly upregulated by TCP5 (Figure 3F), suggesting that these genes might be direct targets of TCP5. We further used electrophoretic mobility shift assays (EMSAs) (Figures 3G and 3H) and chromatin immunoprecipitation quantitative polymerase chain reaction (ChIP-qPCR) (Figures 3I and 3J) to confirm that TCP5 protein could directly bind to the promoter of *YUC8* and *PRE1*. These direct target genes of both TCP5 and PIF4 support the idea that TCP5 controls plant thermomorphogenesis by associating with PIF4.

### TCP5 Controls Plant Thermomorphogenesis by Regulating PIF4 at Transcriptional and Post-transcriptional Levels

**Figure 4:**
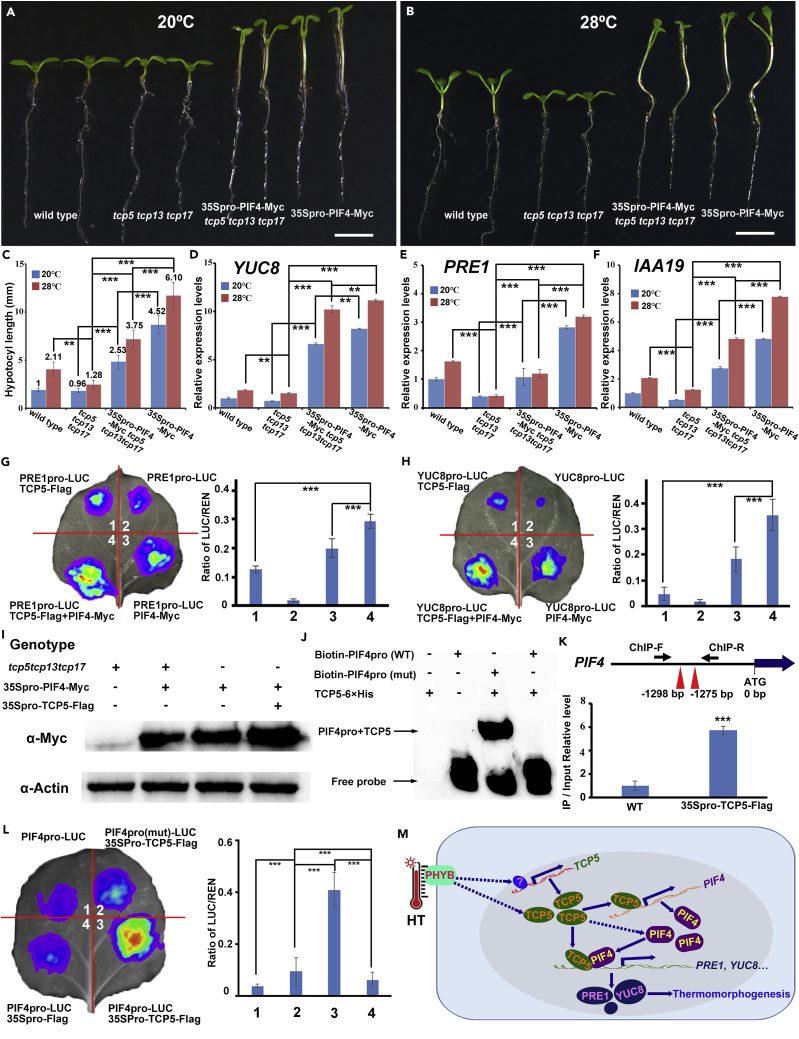
TCP5 Regulates PIF4 Activity at Both the Transcriptional and Post-transcriptional Levels (A and B) The overexpression of PIF4 rescued the aberrant hypocotyl elongation of the *tcp5tcp13tcp17* triple mutant after HT treatment. (C) The hypocotyl length of wild-type, *tcp5 tcp13 tcp17*, 35Spro-PIF4-Myc/*tcp5 tcp13 tcp17,* and 35Spro-PIF4-Myc under 20°C and 28°C. Data represent the mean ± SD of three biological replicates, n > 20. Significant differences are indicated with **p < 0.01 and ***p < 0.001. The number above each column indicates fold changes of hypocotyl length relative to wild-type control under 20°C. (D–F) qRT-PCR assays indicated the transcripts of (D) *YUC8*, (E) *PRE1,* and (F) *IAA19* in plants from (A) and (B). Seven-day-old seedlings grown under 20°C and then transferred to 20°C or 28°C for 2 h before harvesting total RNAs. Data represent the mean ± SD of three biological replicates. Significant differences are indicated with **p < 0.01and ***p < 0.001. (G) Transient dual-luciferase expression in tobacco leaves showed that TCP5, with PIF4, strengthened the activation of *PRE1*. (H) Transient dual-luciferase expression analysis in tobacco leaves indicated that TCP5, with PIF4, strengthened the activation of *YUC8*. (I) The abundance of PIF4 protein in the *tcp5 tcp13 tcp17*, wild-type, or 35Spro-TCP5-FLAG backgrounds. Actin was used as an internal control. This experiment was repeated three times. (J) EMSA showed that TCP5 directly bound the promoter of *PIF4* gene. (K) ChIP-PCR assays showed that TCP5 bound to the promoter region of *PIF4*. 14-day-old wild-type and 35Spro-TCP5-FLAG plants grown under 20°C were collected for ChIP-PCR using the primer pairs as presented in top panel. The data were first normalized to *ACT8* and then were relative to that in input DNA samples. The data represent the mean ± SD of three biological replicates. (L) Transient expression assays demonstrated that TCP5 directly regulated the *PIF4*. The activity of REN was used as an internal control for normalization. The fluorescence ratio of LUC/REN represents the mean ± SD of five biological replicates. Significant differences are indicated with ***p < 0.001 in (G), (H) and (L). (M) A working model of TCPs in the regulation of thermomorphogenesis. HT induces *TCP5* transcription and stabilizes TCP5. The TCP5 that accumulates under HT can directly promote *PIF4* transcription and stabilize PIF4 protein. Moreover, TCP5 enhances PIF4 transactivation activity by interacting with PIF4. Many elongation- and auxin-related genes, such as *YUC8* and *PRE1*, were activated to promote hypocotyl and petiole elongation during thermomorphogenesis. Scale bars, 5 mm in (A and B). See also Figure S4.

To further support the interaction between TCP5 and PIF4, we conducted genetic analyses. We crossed 35Spro-PIF4-Myc with *tcp5 tcp13 tcp17* mutants and 35Spro-TCP5-FLAg with *pif4* mutants. The overexpression of *PIF4* rescued the short hypocotyl of *tcp5 tcp13 tcp17* mutants after HT treatment, whereas the long hypocotyls of the 35Spro-PIF4-Myc were partially rescued by the loss of TCP function in *tcp5 tcp13 tcp17* (Figures 4A–4C). However, we did not observe that the overexpression of *TCP5* could rescue the defects of *pif4* in response to HT (Figures S4A–S4E). We then tested the expression level of *YUC8*, *PRE1,* and *IAA19* coregulated by TCPs and PIF4 in these genetic materials. As predicted, the expression of these genes were downregulated in *tcp5 tcp13 tcp17*, but were upregulated in the overexpression lines of *PIF4* under HT or normal temperature (Figures 4D–4F). These data demonstrate that TCPs and PIF4 synergistically regulate plant thermomorphogenic growth.

To decipher the possible molecular mechanisms by which TCPs promote the function of PIF4, we first tested, based on the data in Figures 3E and 3F, whether TCP5 could enhance the activity of PIF4. We cloned the promoters of *YUC8* and *PRE1*, which are direct target genes coregulated by TCP5 and PIF4. The promoters were used to drive the *LUC* reporter gene in PRE1pro-LUC and YUC8pro-LUC constructs. Transient expression assays in tobacco leaves suggested that the coexpression of PRE1pro-LUC or YUC8pro-LUC with either TCP5 or PIF4 could activate the *LUC* reporter, consistent with the observation that *PRE1* and *YUC8* are direct targets of both TCP5 and PIF4. We then coexpressed PRE1pro-LUC or YUC8pro-LUC with both TCP5 and PIF4, and the expression levels of *LUC* further drastically increased (Figures 4G and 4H), suggesting that TCP5 and PIF4 act together to strengthen the activation of their coregulated genes. We then investigated whether TCP5 could prevent the degradation of PIF4 protein. We crossed the 35Spro-PIF4-Myc plants with 35Spro-TCP5-FLAG plants and *tcp5 tcp13 tcp17* mutant. We tested the abundance of PIF4-Myc protein and found that PIF4-Myc largely accumulated in 35Spro-TCP5-FLAG plant, but showed low abundance in the *tcp5 tcp13 tcp17* mutants (Figure 4I), suggesting that TCPs may have protected the PIF4 protein from degradation. In addition to the promotion of PIF4 stability by TCP5, the expression level of *PIF4* was strongly downregulated in *tcp5 tcp13 tcp17* under HT in our RNA-seq data (Figure S4F). Using qRT-PCR, we confirmed that *PIF4* was indeed highly repressed in *tcp5 tcp13 tcp17* under HT (Figure S4G). To test whether TCP5 might directly regulate *PIF4* gene, we used estradiol to treat pER8-iTCP5 transgenic plants and found that *PIF4* transcripts rapidly increased (Figure S4H). We searched the promoter of *PIF4* and found two possible TCP-binding sites. EMSA analysis and ChIP-qPCR indicated that the two binding sites were required for the direct binding of TCP5 to the promoter of *PIF4*, whereas mutations in the binding site disrupted TCP5 binding to the *PIF4* promoter in EMSA (Figures 4J and 4K). We further used the *PIF4* promoter or a *PIF4-*mutated promoter to drive the *LUC* reporter in PIF4pro-LUC or PIF4pro(mut)-LUC. Transient expression assays confirmed that TCP5 could directly bind to the promoter of *PIF4* so that *PIF4* expression could be activated (Figure 4L). These data suggest that TCP5 promotes the activity of PIF4 at both transcriptional and post-translation levels.

In this study, we demonstrate that TCP5 acts as an important component in the control of plant thermomorphogenesis by boosting PIF4 activity. We showed that *TCP5* is rapidly induced by HT and that HT stabilizes TCP5 proteins. The overexpression of *TCP5* causes constitutive thermomorphogenesis under normal growth temperature, whereas thermomorphogenesis is compromised in the *tcp5 tcp13 tcp17* triple mutant. We propose a working model for the regulation of plant thermomorphogenesis by TCP5 (Figure 4M). HT promotes the transcription of *TCP5* and the stability of TCP5 protein. On the one hand, TCP5 directly binds the *PIF4* promoter and upregulates the transcriptional level of *PIF4*; on the other hand, TCP5 directly interacts with PIF4 to promote the stability and activity of PIF4 protein. TCP5 and PIF4 coactivate many HT-inducible genes to stimulate the growth of hypocotyls and petioles. Our data demonstrate that TCP5 acts as a positive regulator of plant response to HT. TCPs control the biosynthesis of multiple plant hormones (Barkoulas et al., 2007, Challa et al., 2016, Danisman, 2016, Gonzalez-Grandio et al., 2017, Guo et al., 2010, Lopez et al., 2015, Nicolas and Cubas, 2016, Schommer et al., 2008, Steiner et al., 2012, Wang et al., 2015), including auxin (Challa et al., 2016, Koyama et al., 2010), BRs (Guo et al., 2010), jasmonic acid (Schommer et al., 2008), salicylic acid (Wang et al., 2015), and abscisic acid (Gonzalez-Grandio et al., 2017). Our findings suggest that TCPs might function as important relays that convert environmental cues such as HT into endogenous signals during plant morphological response to ever-changing environments.

### Limitations of the Study

In this study, we find that a clade of TCP transcription factors that include TCP5, TCP13, and TCP17 act as important positive regulators by boosting PIF4 activity at both transcriptional and protein levels during thermomorphogenesis. Although this study provides a mechanism for fine-tuning PIF4 activity during plant responses to HT, the relationships between TCPs and other PIF4 interactors including BZR1 and phyB are still unknown. The coordination of TCPs with other numerous regulators in the tight control of PIF4 activity and plant thermomorphogenesis needs to be subsequently studied in future.

## Methods

All methods can be found in the accompanying Transparent Methods supplemental file.

## Supplemental Information

Document S1. Transparent Methods, Figures S1–S4, and Tables S4

Table S1. RNA-Seq Analysis of Regulated Genes after HT Treatment in the Leaf Petioles or Blades of Wild-Type, Related to Figure 3

Table S2. RNA-Seq Analysis of DEGs Regulated by PIF4 or TCPs, Related to Figure 3

Table S3. RNA-Seq Analysis of Genes Co-upregulated by PIF4 and HT in the Petioles, Related to Figure 3
