# Supplementary material for: Arabidopsis Transcription Factor TCP5 Controls Plant Thermomorphogenesis by Positively Regulating PIF4 Activity
Source: iScience. 2019 May 8;15:611–22. doi: 10.1016/j.isci.2019.04.005 (PMC6548983; doi:10.1016/j.isci.2019.04.005)
Supplement: Document S1. Transparent Methods, Figures S1–S4, and Tables S4 [file mmc1.pdf]

ISCI, Volume *15*

Supplemental Information

Arabidopsis Transcription Factor TCP5 Controls Plant Thermomorphogenesis by Positively Regulating PIF4 Activity

Xiang Han, Hao Yu, Rongrong Yuan, Yan Yang, Fengying An, and Genji Qin

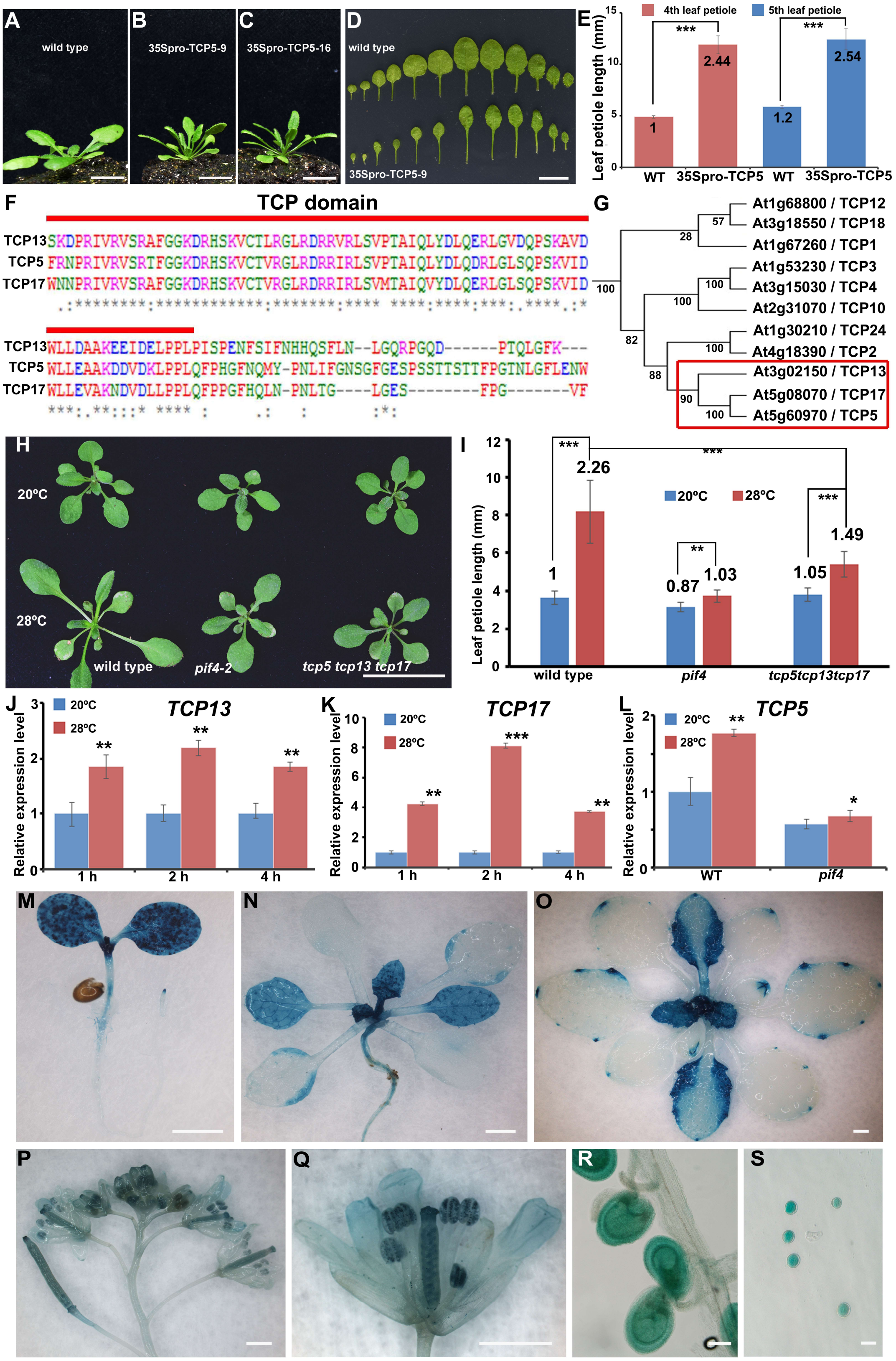

Figure S1. TCP5 and its close homologs participate in plant thermomorphogenesis, Related to Figure 1.

(A-C) 21-day-old wild-type (A), 35Spro-TCP5-Flag-9 (B) and 35S-TCP5- FLAG-16 (C) plants. The leaves grow upward in 35Spro-TCP5-Flag at 20°C. (D) Dissected leaves of 21-day-old wild-type and 35Spro-TCP5-Flag-9 plants at 20°C. (E) Quantitative analysis of length of the 4th and 5th petioles from wild type and 35Spro-TCP5-Flag-9. Significant differences are indicated with *** ($P < 0.01$, n>20, two-tailed Student′s t-test). The number above each column means fold changes of petiole length relative to wild type. (F) Protein alignment assays showing that TCP5, TCP13 and TCP17 share a conserved noncanonical bHLH domain. (G) Phylogenetic analysis of the full lengths of the class II TCP family in Arabidopsis. TCP5, TCP13 and TCP17 are grouped in a small subclade, suggesting that they have high similarity. The phylogenetic tree was constructed using MEGA 6.0 with default settings. (H) 26-day-old wild type, *pif4* mutant and *tcp5tcp13tcp17* mutant. 21-day-old plants grown in continuous 20°C before transferring to 20°C or 28°C for 5 additional days of growth. (I) Quantitative analysis of the length of the 5th petiole of wild type, *pif4* mutant and *tcp5tcp13tcp17* mutant. The data represent the mean ± SD of three biological replicates. Significant differences are indicated with * ($P < 0.05$, n>20) and *** ($P < 0.001$). The number above each column means fold changes of hypocotyl length relative to that of wild type under 20°C. (J) and (K) Relative expression levels of *TCP13* (J) and *TCP17* (K) in wild type after HT treatment for 1, 2 or 4 hours. The expression levels were normalized to that of *ACT8* and were relative to that of wild-type control under 20°C. Data represent the mean ± SD of three biological replicates. (L) The expression changes of TCP5 in *pif4* mutant under 20°C or 28°C. (M) GUS staining of 10-day-old TCP5pro-GUS transgenic seedlings. (N) GUS staining of 21-day-old TCP5pro-GUS transgenic plants. (O) GUS staining of 28-day-old TCP5pro-GUS transgenic plants. (P) GUS staining of inflorescence from 35-day-old TCP5pro-GUS transgenic plants. (Q) GUS staining of flowers from 35-day-old TCP5pro-GUS transgenic plants. (R) GUS staining of ovules from 35-day-old TCP5pro-GUS transgenic plants. (S) GUS staining of pollen grains from 35-day-old TCP5pro-GUS transgenic plants. Scale bars, 1 cm in (A-C), 1.5 mm in (D), 2 cm in (H), 1 mm in (M-Q), 50 μm in (R) and (S).

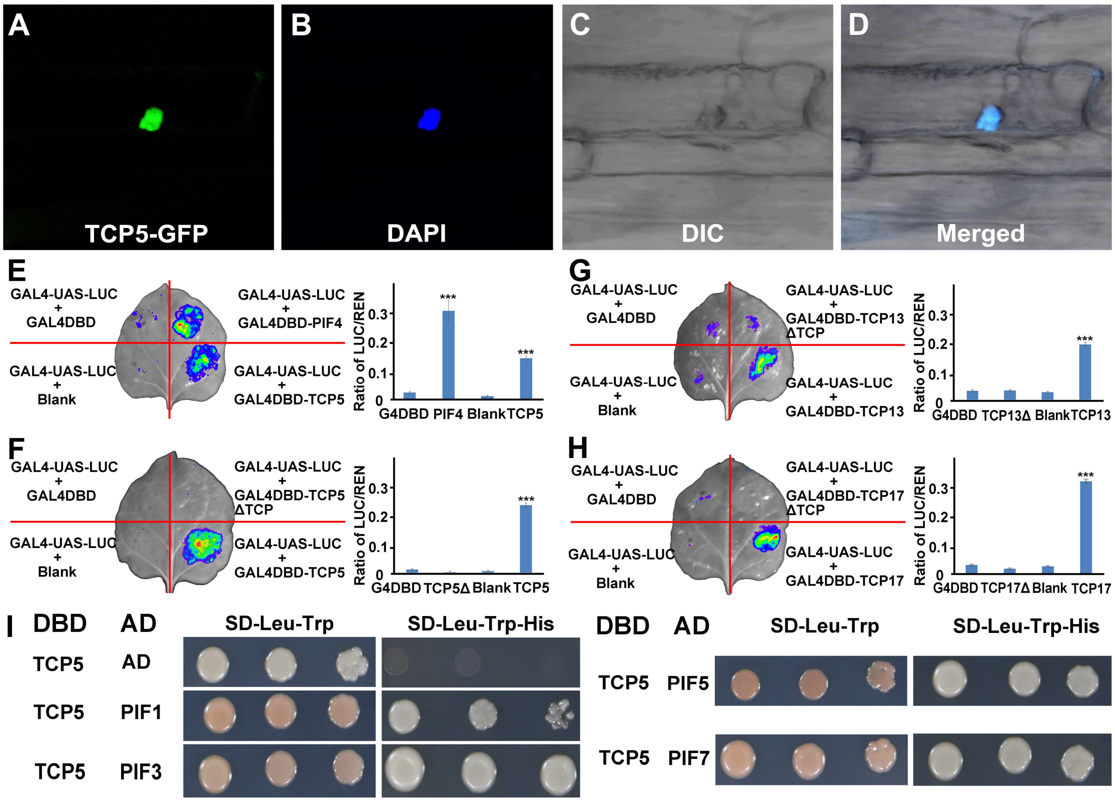

Figure S2. The subcellular localization of TCP5 and the transactivation activity of TCP5, TCP13 and TCP17, Related to Figure 2.

(A) Green fluorescence detected in the hypocotyls of TCP5pro-TCP5-GFP seedlings. (B) Nuclei stained with DAPI. (C) Bright field image of TCP5pro-TCP5-GFP hypocotyl. (D) Image merge of DAPI and GFP. (E) PIF4 and TCP5 act as transactivators. (F-H) TCP5, TCP13 and TCP17 work as transactivators, and their transactivation activity is dependent on the bHLH domains of TCP5 (F), TCP13 (G) and TCP17 (H). (I) Yeast two hybrid assays showed that TCP5 interacted with PIF1, PIF3, PIF5 and PIF7. Co-transformed yeast cells were grown on medium lacking Leu and Trp (SD-Leu-Trp) and selected on medium lacking Leu, Trp and His (SD-Leu-Trp-His) with or without 10 mM 3-AT at dilutions of 10 and 100 fold. The empty vector pDEST22 was used as a negative control. Scale bars, 10 μm in (A-D).

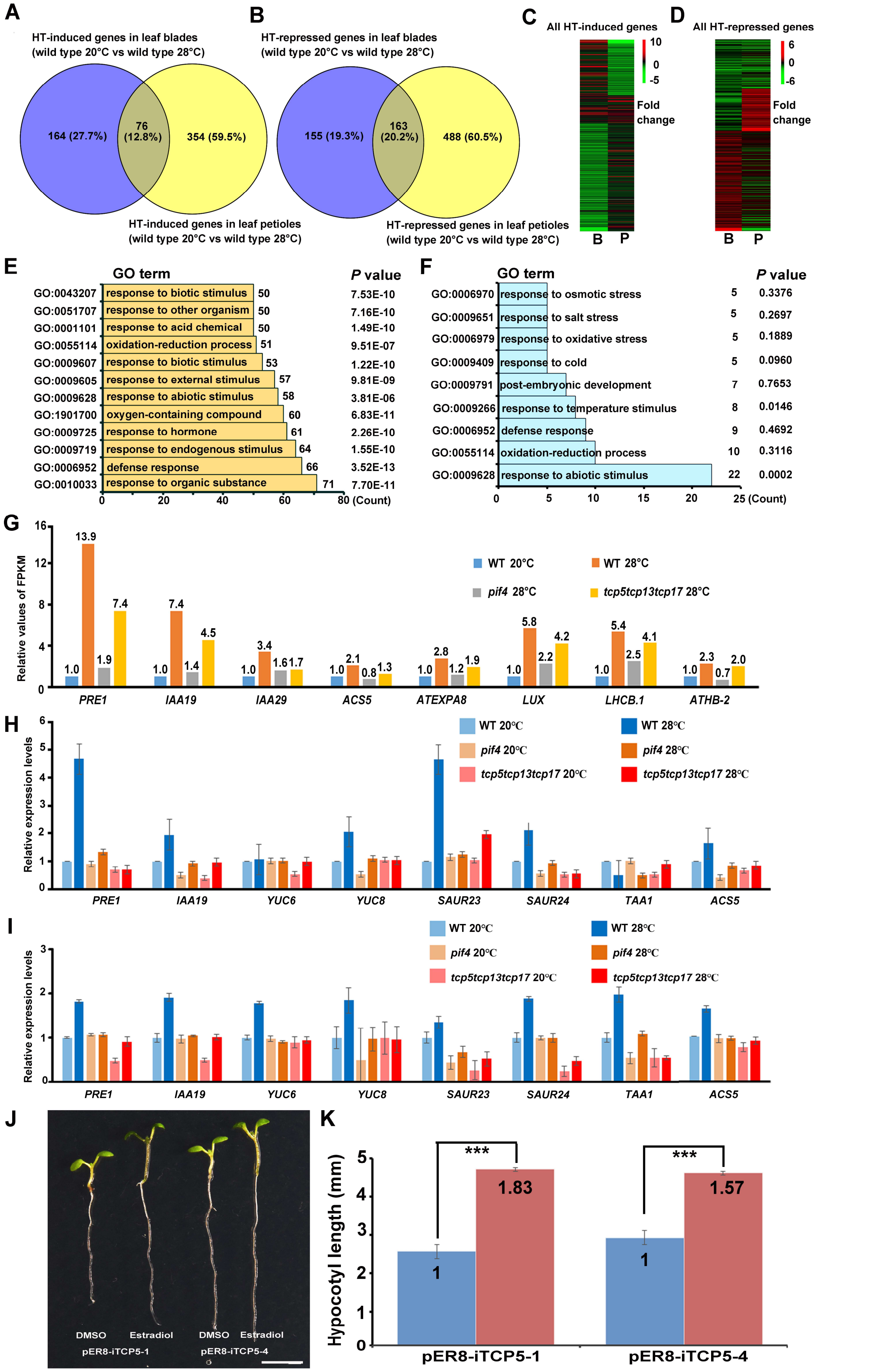

Figure S3. Transcriptomes regulated by HT in petioles and leaf blades, and the target genes regulated by TCPs, Related to Figure 3.

(A) Venn diagrams of genes upregulated by HT in petioles and leaf blades. (B) Venn diagrams of genes downregulated by HT in petioles and leaf blades. (C) Heat map of genes upregulated by HT in petioles and leaf blades. (D) Heat map of genes downregulated by HT in petioles and leaf blades. Scale bars indicate the fold changes of the DEGs. (E) GO analysis of genes up-regulated by both PIF4 and TCPs in petioles. (F) GO analysis of down-regulated by both PIF4 and TCPs in petioles. GO analysis were performed by DIVID 6.8 with default settings. (G) Diagrams of relative FPKM values of PIF4 direct target genes from RNA-seq data in WT at 20°C and 28°C, *pif4* at 28°C and *tcp5tcp13tcp17* at 28°C. (H) qRT-PCR analysis of the expression levels of TCPs- and PIF4-coregulated genes from hypocotyls. 7-day-old wild-type, *pif4* mutant and *tcp5tcp13tcp17* mutant seedlings grown under continuous 20°C before transferring to 28°C for additional 2 hours. *ACT8* was used as an internal control. (I) qRT-PCR analysis of the expression levels of TCPs- and PIF4-coregulated genes from petioles. 21-day-old, *pif4* and *tcp5tcp13tcp17* mutant grown in continuous 20°C before transferring to 28°C for additional 2 hours. *ACT8* was used as an internal control. (J) Estradiol- or mock-induced pER8-iTCP5-1 and pER8-iTCP5-4 transgenic plants. 10-day-old seedlings which were first grown for 3 days and then treated with 50 μM estradiol or DMSO for 7 days under 20°C. (K) Quantitative analysis of the hypocotyl lengths of pER8-iTCP5-1 and pER8-iTCP5-4 in (J). These data represent the mean ± SD of three biological replicates. Significant differences are indicated with *** ($P < 0.001$, n>20). The number above each column means fold changes of hypocotyl length relative to mock treatment. Scale bar=5 mm in (J).

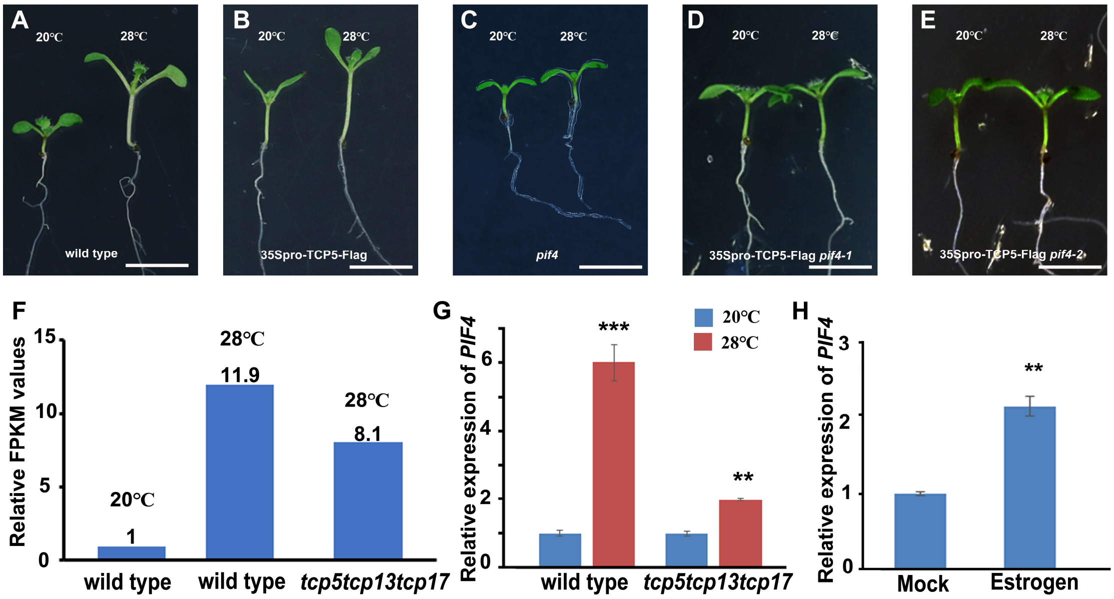

Figure S4. Genetic and regulation analysis between TCP5 and PIF4. Related to Figure 4.

(A-E) Overexpression of *TCP5* could not rescue the insensitivity of *pif4* mutant in response to HT. The hypocotyl of wild type (A), *pif4* (B), 35Spro-TCP5-Flag (C) and 35Spro-TCP5-Flag *pif4*-1 (D) and 35Spro-TCP5-Flag *pif4*-2 (E) under 20°C and 28°C. (F) Diagrams of relative FPKM values of *PIF4* gene from RNA-seq data in wild type at 20°C, wild type treated with 28°C, *tcp5tcp13tcp17* treated with 28°C. (G) qRT-PCR analysis of the expression level of *PIF4* gene from hypocotyls. 7-day-old wild-type and *tcp5tcp13tcp17* seedlings grown under continuous 20°C before transferring to 28°C for 2 additional hours. *ACT8* was used as an internal control. The data represent the mean ± SD of three biological replicates. (H) qRT-PCR analysis of *PIF4* in TCP5-inducible pER8-iTCP5 transgenic plants. The pER8-iTCP5 transgenic plants were treated with 50 µM estradiol or DMSO for 2 hours. The gene expression levels were normalized to that of *ACT8* and are relative to that in pER8-iTCP5 treated with DMSO. The data represent the mean ± SD of three biological replicates.

Table S4. The list of oligonucleotides used in this study. Related to Figure 1, 2, 3 and 4.

Oligonucleotide name	sequence (5' - 3')
**Oligonucleotides for constructs**	
PIF4-F	CACCATGGAACACCAAGGTTGG
PIF4-R-NSC	TCCGTGGTCCAAACGAGAACCGTC
PIF4-R-SC	CTATCCGTGGTCCAAACGAGAACCGTC
TCP5-F	CACCATGAGATCAGGAGAATGTGATGAAGAG
TCP5-R-NSC	AGAATCTGATTCATTATCGC
TCP5-R-SC	TCAAGAATCTGATTCATTATCGC
TCP5-F1	CCGCTCGAGATGAGATCAGGAGAATGTGA
TCP5-R1	GGACTAGTTCAAGAATCTGATTCATTAT
TCP13-F	CACCATGAATATCGTCTCTTGGAAAGATGC
TCP13-R-NSC	CATATGGTGATCACTTCCTCTA
TCP13-R-SC	TCACATATGGTGATCACTTCCTCTA
TCP17-F	CACCATGGGAATAAAAAAAGAAGATCA
TCP17-R-NSC	CTCGATATGGTCTGGTTGTGAG
TCP17-R-SC	CTACTCGATATGGTCTGGTTGTGAG
TCP5pro-F1	CACCTGCCAAATCCTGTCATACATTCCCAA
TCP5pro-R1	CTCTTTAATTCACTCAACAAGATCTTCA
TCP5pro-F2	GGGGACAACTTTGTATAGAAAAGTTGCTGCCAAATCCTGTCATA
TCP5pro-R2	GGGGACTGCTTTTTTGTACAAACTTGCCTCTTTAATTCACTCAAC
YUC8pro-F	GGGGACAACTTTGTATAGAAAAGTTGCTAAAGTGTCTAGATGA
YUC8pro-R	GGGGACTGCTTTTTTGTACAAACTTGCTAAGTTTCTTTAATAAGT
PRE1pro-F	GGGGACAACTTTGTATAGAAAAGTTGCTTCATGCAAACCTGTCG
PRE1pro-R	GGGGACTGCTTTTTTGTACAAACTTGCCATGTTCAACAATGTGG
PIF4pro(wt)-F	GGGGACAACTTTGTATAGAAAAGTTGCTACACCATCACGAATTT
PIF4pro(wt)-R	GGGGACTGCTTTTTTGTACAAACTTGCTAGCGTTATGGTTTTTGT
PIF4pro(mut)-F	GGATATGGGTGATTACAAGTAGGCACAATGGGTGACTAAT
PIF4pro(mut)-R	ATTAGTCACCCATTGTGCCTACTTGTAATCACCCATATCC
**Oligonucleotides for yeast two hybrid**	
PIF4△N53-F	CACCATGGATCATCATGAAGAAGCCCTAAGAT
PIF4△C-R	TTAATGAACTTCAGCTGCTCGACTCCTT
PIF4△N-F	CACCATGATGTGGATGGGGAGTGGAATGGCG
PIF4-bHLH-F	CACCATGAATCTCTCCGAAAGGAGGAGG
PIF4-bHLH-R	TTACACTTGAAGCTGTAACTGAAGTGA
TCP5△N1-F	CACCATGCCTCCTCTACAATTCCCACATGGATTTAAC
TCP5△TCP-R	GAATGTTCTTGAGACTCGAACGATTCTTGGATTC
TCP5△TCP-F	GAATCCAAGAATCGTTCGAGTCTCAAGAACATTC
TCP5△N2-F	CACCATGGGTGGCAAAGACAGACACAGCAAAG
**Oligonucleotides for genotyping**	

TCP5-SM-LP	TGAATCTGTTTTTCCTCCATCC
TCP5-SM-RP	CTCGAAGCAGCAAAAGATGAC
TCP13-SM-LP	TTCCTGTTCAAGCTCAAGACC
TCP13-SM-RP	GACCGACGACATCCGATTATC
TCP17-SALK-LP	TCTTTGGATCCTCAGATCTTCC
TCP17-SALK-RP	ATGTACCTTTGCTCGCATCAG
PIF4-SALK-LP	AATTCATCATCGGGGATTAGG
PIF4-SALK-RP	TCGTCGTTTAATAAACACGGC
SM-P	TACGAATAAGAGCGTCCATTTTAGAGTGA
SALK-P	ATTTTGCCGATTTCGGAAC
**Oligonucleotides for qRT-PCR**	
ACTIN8-qRT-F	TGTGCCAATCTACGAGGGTTT
ACTIN8-qRT-R	TTTCCCGCTCTGCTGTTGT
PIF4-qRT-F	CCAGATCATCTCCGACCGGTTTG
PIF4-qRT-R	CTAGTGGTCCAAACGAGAACCGT
YUC6-qRT-F	TGGCAGCTCAAGACTTATGACCGT
YUC6-qRT-R	TCCGGCTTTATGTCAAACCTCCGA
YUC8-qRT-F	TGTATGCGGTTGGGTTTACGAGGA
YUC8-qRT-R	CCTTGAGCGTTTCGTGGGTTGTTT
PRE1-qRT-F	GTTCTGATAAGGCATCAGCCTCG
PRE1-qRT-R	CATGAGTAGGCTTCTAATAACGG
PRE2-qRT-F	CCGTCGTTCCAACACGGTATCA
PRE2-qRT-R	CTGCGGCTTGTGGGCTATTAGG
IAA19-qRT-F	GGTGACAACTGCGAATACGTTACCA
IAA19-qRT-R	CCCGGTAGCATCCGATCTTTTCA
IAA29-qRT-F	CACGGCGATGAACAACAACATAT
IAA29-qRT-R	CTCTGTCGCAATCTTCATATTCG
ACS5-qRT-F	GCGATGCTTTCCTTTTGCCTACTC
ACS5-qRT-R	TTTCTGGGCTTGTTGGTAAGCTTGT
SAUR23-qRT-F	ATTCAAACTTTCAGACAAAAGAAATGG
SAUR23-qRT-R	ACAAGGAAACAACTCTATCTCTAACT
SAUR24-qRT-F	GAGATATTTGGTGCCTGTCTCATATTTAAACC
SAUR24-qRT-R	CAAGAAGAAAGAGGAAAAAGGGCTCATC
TAA1-qRT-F	CCCTGCGTTTGCGTGGCTAGGGA
TAA1-qRT-R	GAGCTTCATGTTGGCGAGTCTCT
TCP5-qRT-F	GGGTTTAACACCAATCATCAACAA
TCP5-qRT-R	CGACAGTAACGTTATTACCAGATT
TCP13-qRT-F	CTGGTTCAGGGACTATGGAGACATT
TCP13-qRT-R	AAATGTTTTGGGAAGACGAAGATGA
TCP17-qRT-F	GGTAACGTCACTGTCGCATTTTCTAA
TCP17-qRT-R	GAAACGAAGGGTACCTTGTTGGGA

**Oligonucleotides for EMSA**	
TCP5-6*HIS-F	CACCTCTAGAATGATATTTAGGTGACACTATAGAACAGACCACC
TCP5-6*HIS-R	CTCGAGAAAAAAAAAAAAAAAAAATTAATGGTGATGGTGATGA
PIF4-EMSA-F(+3'	ATGGGATATGGTCCATTACAAGTAGGCACAATGGTCCACTAAT
PIF4-EMSA-R	ATAACAATTATTAGTGGACCATTGTGCCTACTTGTAATGGACCA
PIF4-EMSA-mut-F(+3'	ATGGGATATGGGTGATTACAAGTAGGCACAATGGGTGACTAAT
PIF4-EMSA-mut-R	ATAACAATTATTAGTCACCCATTGTGCCTACTTGTAATCACCCA
YUC8-EMSA-F(+3'	TCTTCCCCACGTGGCTTCCTCTCGTTGGTCCCACAAAGAAT
YUC8-EMSA-R	ATTCTTTGTGGGACCAACGAGAGGAAGCCACGTGGGGAAGA
YUC8-EMSA-mut-	TCTTCCCCACGTGGCTTCCTCTCGTTGGGTGCACAAAGAAT
YUC8-EMSA-mut-R	ATTCTTTGTGCACCCAACGAGAGGAAGCCACGTGGGGAAGA
PRE1-EMSA-F(+3'	ATGGTGGTGACACGTGACATAGTCGATGTTTTGTGAACATTTAT
PRE1-EMSA-R	ATTGTTCGATTGGTCCATTATTGGTTGAGTATTAATTTATATAAA
PRE1-EMSA-mut-	ATGGTGGTGACACGGGACATAGTCGATGTTTTGTGAACATTTAT
PRE1-EMSA-mut-R	ATTGTTCGATTGGGTGATTATTGGTTGAGTATTAATTTATATAA
	**Oligonucleotides for ChIP-PCR**
YUC8-Y1-F	ATGTGGAGATGGCGGGACCAAGAAA
YUC8-Y1-R	GGGTGATTCTTTGTGGGACCAACGA
PRE1-P1-F	CAGCATGTAACACGAGACATGGTGG
PRE1-P1-R	CGGCTTTAAAACTTCGTTCAGACCA
PRE1-P2-F	ACACAATAACTAACTAACCATGATTGAAAACG
PRE1-P2-R	AATAAACCTCGACAATGATGTCACG
PIF4-ChIP-F	TGATATACATTTCAGGAAAACTTAGC
PIF4-ChIP-R	GGAAAAAGATCTAGTATGTTCTTCG

Transparent Methods.

KEY RESOURCES TABLE

REAGENT or RESOURCE	SOURCE	IDENTIFIER
Antibodies		
Mouse anti-MYC antibody	CMCTAG	Cat. AT0023
Mouse anti-FLAG antibody with HRP conjugating	Sigma-Aldrich	Cat. A8592
Mouse anti-plant actin antibody	Sigma-Aldrich	Cat. A0480
EZview™ red anti-c-Myc affinity gel	Sigma-Aldrich	Cat. E6654
Anti-FLAG® M2 Magnetic Beads	Sigma-Aldrich	Cat. M8823
Goat anti-mouse HRP-conjugated IgG	CWBIO	Cat. Cw0102
Bacterial and Virus Strains		
E.coli strain DH5α	N/A	N/A
A. tumefaciens GV3101	N/A	N/A
Yeast strain AH109	N/A	N/A
Chemicals, Peptides, and Recombinant Proteins		
β-Estradiol	Sigma-Aldrich	Cat. E2758
DAPI (4',6-diamidino-2-phenylindole dihydrochloride)	VECTOR	Cat. H-1200
3-AT (3-amino-1,2,4 triazole)	Sigma-Aldrich	Cat. A9126
D-Luciferin	Sigma-Aldrich	Cat. L0594
X-gluc (5-Bromo-4-chloro-3-indolyl β-D-glucuronide)	Sigma-Aldrich	Cat. B5285
Critical Commercial Assays		
First-strand cDNA reverse transcription kit	Promega	Cat. A3500
Dual-luciferase reporter kit	Promega	Cat. E1910
TNT SP6 high-yield wheat germ protein expression kit	Promega	Cat. L3260
pENTR/D-TOPO Cloning Kit	Thermo Fisher	Cat. K240020
Gateway LR Clonase II Enzyme mix	Thermo Fisher	Cat. 11791020
LightShiftTM Chemiluminescent EMSA kit	Thermo Fisher	Cat. 20148
Experimental Models: Organisms/Strains		
Arabidopsis: WT Col-0	N/A	N/A
Arabidopsis: *tcp5*	[29]	SM_3_29639
Arabidopsis: *tcp13*	[29]	SM_3_23151
Arabidopsis: *tcp17*	[29]	SALK_147288
Arabidopsis: *pif4*	[12]	SALK_140393C
35Spro-PIF4-MYC/*tcp5tcp13tcp17*	This study	N/A
35Spro-TCP5-FLAG/*pif4*-1	This study	N/A

35Spro-TCP5-FLAG/*pif4*-2	This study	N/A
35Spro-PIF4-MYC/35Spro-TCP5-FLAG	This study	N/A
Recombinant DNA		
TCP5pro-GUS	This study	N/A
TCP5pro-TCP5-GFP	This study	N/A
35Spro-PIF4-Myc	This study	N/A
35Spro-TCP5-Flag	This study	N/A
pER8-iTCP5	This study	N/A
35Spro-TCP13-Flag	This study	N/A
35Spro-TCP17-Flag	This study	N/A
PIF4pro-LUC	This study	N/A
PIF4mpro-LUC	This study	N/A
PRE1pro-LUC	This study	N/A
YUC8pro-LUC	This study	N/A
Software and Algorithms		
ImageJ	N/A	https://imagej.net/ Welcome
Excel (2016)	Microsoft Office Excel	N/A
Photoshop (cc2018)	Adobe	N/A
MEGA (6.0)	N/A	https://www.megasoftware.net/
DIVID (6.8)	N/A	https://david.ncifcrf.gov/
BMK Cloud	Biomarker Technology Co.	https://www.biocloud.net/

EXPERIMENTAL MODEL AND SUBJECT DETAILS

Plant materials and growth conditions

The *Arabidopsis thaliana* ecotype Columbia-0 (Col-0) was used as the wild type in this study. T-DNA mutants and various transgenic plants were in the Col-0 background. The T-DNA insertion mutants were *tcp5*, *tcp13*, *tcp17* (Efroni et al., 2008) and *pif4* (Zhang et al., 2018). The transgenic plants are described below. Wild-type, mutant and transgenic seeds were first sterilized using 15% (v/v) sodium hypochlorite solution and then plated on half-strength Murashige and Skoog medium, which was supplemented with 20 µg/mL DL-phosphinothricin, 50 µg/mL kanamycin or 50 µg/mL hygromycin B if necessary. The seeds were then synchronized at 4°C for 3 days and then placed in a 20±1°C growth chamber with long-day (16-hour light and 8-hour dark) conditions for 7 days. Green seedlings were transferred to soil and put in a 20±1°C greenhouse or growth chamber under long-day conditions. *Nicotiana benthamiana* was grown in soil and put in the same greenhouse for transient expression assays. For high-temperature

treatment, 7-day-old seedlings or 21-day-old plants were transferred to a 28±1°C growth chamber for 3 days or 5 days.

Accession numbers

Gene annotations and data in this article can be found in The Arabidopsis Information Resource (TAIR) database using the following accession numbers: TAIR: AT5G60970 (TCP5), TAIR: AT3G02150 (TCP13), TAIR: AT5G08070 (TCP17), TAIR: AT2G43010 (PIF4), TAIR: AT5G39860 (PRE1), and TAIR: AT4G28720 (YUCCA8).

METHOD DETAILS

PCR analysis and gene expression assays

All the primers used in this study are listed in Table S4.

The T-DNA insertion mutants were genotyped using the primers TCP5-SM-LP, TCP5-SM-RP and SM-P; TCP13-SM-LP, TCP13-SM-RP and SM-P; TCP17-SALK-LP, TCP17-SALK-RP and SALK-P; or PIF4-SALK-LP, PIF4-SALK-RP and SALK-P for *tcp5*, *tcp13*, *tcp17* and *pif4*, respectively. PCR was performed for 30 cycles (94°C for 20 s, 52-60°C for 20 s, and 72°C for 90 s).

To perform qRT-PCR, total RNA from seedlings, petioles or blades were extracted by TRIzol reagent (Invitrogen) and reverse-transcribed using reverse transcription kit (Promega) according to the manufacturer's instructions. The products were used as the templates for qRT-PCR, which was conducted in an Applied Biosystems 7500 Fast Real-time PCR system with UltraSYBR Mixture (CWBIO, CW2601) as described in the manual. The PCR conditions were 95°C for 10 min, then performed 40 cycles under conditions of 94°C for 10 s, 58-60°C for 15 s, and 72°C for 25 s. Each experiment was repeated three times. *ACT8* was used as an internal control. The relative expression levels were evaluated via the $2^{-\Delta\Delta CT}$ (cycle threshold) method (Livak and Schmittgen, 2001).

Generation of binary constructs and transgenic plants

To examine the expression pattern of *TCP5*, the 2400-bp promoter upstream of the *TCP5* start codon was amplified using primers TCP5pro-F1 and TCP5pro-R1 and then cloned into pENTR/D-TOPO (Invitrogen) to generate TCP5pro-TOPO. The TCP5pro-GUS vectors were generated via an LR reaction with TCP5pro-TOPO and pKGWFS7 (Ghent University).

To determine the subcellular localization of TCP5, the 2400-bp promoter upstream of the *TCP5* start codon was amplified using primers TCP5pro-F2 and TCP5pro-R2 and then cloned into pDONR-P4P1R (Invitrogen) to generate TCP5pro-P4P1R. And the coding region of *TCP5* without stop codon was amplified using primers TCP5-F and TCP5-R-NSC and then cloned into pENTR/D-TOPO (Invitrogen) to generate TCP5NSC-TOPO. The *GREEN FLUORESCENT PROTEIN* (*GFP*) gene was amplified

from pK7WGF2 (Ghent University) using primers GFP-F and GFP-R and then cloned into pDONR-P2R-P3 (Invitrogen) to generate GFP-P2RP3. TCP5pro-TCP5-GFP was generated via an LR reaction with TCP5pro-P4P1R, TCP5NSC-TOPO, GFP-P2RP3 and pK7m34GW (Ghent University).

To obtain the *PIF4* and *TCP5* overexpression lines, *PIF4* without stop codon was amplified with PIF4-F and PIF4-R-NSC and cloned into pENTR/D-TOPO to generate PIF4NSC-TOPO. The 35Spro-PIF4-Myc or 35Spro-TCP5-Flag constructs were generated via an LR reaction with PIF4NSC-TOPO and pK7MYCGW2 (Li et al., 2016) or with TCP5NSC-TOPO and pK7FLAGGW2 (Li et al., 2016).

To obtain estrogen-inducible *TCP5* transgenic plants, *TCP5* was amplified using primer TCP5-F1, which had an *Xho* I *site,* and primer TCP5-R1, carrying a *Spe* I site. The PCR products and pER8 vectors were digested by *Xho* I and *Spe* I. The pER8-iTCP5 vectors were generated by ligating *Xho* I-*Spe* I-digested PCR products with the *Xho* I-*Spe* I-digested pER8 vector.

To generate 35Spro-TCP13-Flag and 35Spro-TCP17-Flag, *TCP13* and *TCP17* were amplified using primers TCP13-F and TCP13-R-NSC and primers TCP17-F and TCP17-R-NSC, respectively. The two genes were cloned into pENTR/D-TOPO to generate TCP13NSC-TOPO and TCP17NSC-TOPO. The 35Spro-TCP13-Flag and 35Spro-TCP17-Flag constructs were generated via an LR reaction between TCP13NSC-TOPO or TCP17NSC-TOPO and pK7FLAGGW2.

To test TCP5 binding to the *PIF4* promoter, the *PIF4* promoter was amplified using primers PIF4pro(wt)-F and PIF4pro(wt)-R and then cloned into pDONR-P4P1R to generate PIF4pro-P4P1R. pENTR/D-FLUC was generated as described previously (Guo et al., 2015). The PIF4pro-LUC reporter was generated via LR reactions with PIF4pro-P4P1R, pENTR/D-FLUC and pH7m24GW (Ghent University). To generate the *PIF4* mutated promoter, mutations in the *cis*-element (5′-TGGTCC-3′ mutated to 5′-TGGGTG-3′) were included in primers PIF4pro(mut)-R and PIF4pro(mut)-F. PIF4pro(wt)-F and PIF4pro(mut)-R or PIF4pro(mut)-F and PIF4pro(wt)-R were used to amplify the two halves of the *PIF4* mutated promoter. The two halves were then mixed together and used as PCR templates with primers PIF4pro(wt)-F and PIF4pro(wt)-R to obtain the *PIF4* mutated promoter. The *PIF4* mutated promoter was cloned into pDONR-P4P1R to generate PIF4mpro-P4P1R. The PIF4mpro-LUC reporter was generated via an LR reaction with PIF4mpro-P4P1R, pENTR/D-FLUC and pH7m24GW.

To generate PRE1pro-LUC and YUC8pro-LUC, the *PRE1* and *YUC8* promoters were amplified using *PRE1*pro-F and *PRE1*pro-R or *YUC8*pro-F and *YUC8*pro-R. The fragments were cloned into pDONR-P4P1R to generate PRE1pro-P4P1R or YUC8pro-P4P1R. The PRE1pro-LUC and YUC8pro-LUC reporter vectors were generated via an LR reaction with PRE1pro-P4P1R or YUC8pro-P4P1R, pENTR/D-FLUC and pH7m24GW.

The binary constructs were transformed into Agrobacterium strain GV3101 by electroporation. Transgenic Arabidopsis were generated using the floral dip method.

For genetic analysis, 35Spro-PIF4-Myc *tcp5tcp13tcp17* was obtained by crossing 35Spro-PIF4-Myc with *tcp5tcp13tcp17*. The 35Spro-PIF4-Myc 35Spro-TCP5-Flag line was obtained by crossing 35Spro-PIF4-Myc with 35Spro-TCP5-Flag.

Staining and microscopy

Tissues from TCP5pro-GUS lines were fixed in 90% acetone overnight at 4°C and then washed three times using 1× phosphate buffer (pH 6.0). The samples were then immersed into GUS staining buffer solution (1 mg/mL X-gluc in phosphate buffer) and placed under vacuum for 2 hours. The samples were kept in a 37°C incubator for 12~24 hours and then transferred into 75% ethanol to stop staining before observation.

To observe subcellular localization, 7-day-old TCP5pro-TCP5-GFP seedlings were treated with DAPI staining solution for 5-10 min and observed using a Leica TCS SPE confocal microscope. The GFP signal was observed at 488 nm, and the DAPI signal was observed at 340 nm.

The measurement of hypocotyl and petiole lengths

Pictures of hypocotyls or petioles were first taken using a Canon digital camera, and their lengths were then measured using ImageJ software. To analyze hypocotyl elongation under HT, 7-day-old seedlings growing at 20°C were transferred to 28°C for further growing 3 days before being photographed. To analyze petiole elongation under HT, 21-day-old plants growing under 20°C were transferred to 28°C for further growing 5 days before being photographed. The experiments were repeated three times. The data were then analyzed using Microsoft Excel, and statistical significance was determined using Student's t test ($n > 20$).

Yeast two-hybrid assays

To test the PIF4 and TCPs interaction, prey constructs PIF4-AD or TCPs-AD were generated via LR reactions between PIF4-TOPO or TCPs-TOPO and pDEST22 (Invitrogen). To generate the bait constructs, a truncated PIF4Δ53 with the first 53 amino acids of the N-terminus deleted was used because full-length PIF4 can activate the reporter in yeasts. PIF4Δ53 was amplified from PIF4-TOPO plasmids using primers PIF4Δ53-F and PIF4-R-SC and cloned into pENTR/D-TOPO to generate PIF4Δ53-TOPO. The bait constructs DBD-PIF4Δ53 and DBD-TCPs were generated via an LR reaction between PIF4Δ53-TOPO or TCP-TOPO and pDEST32 (Invitrogen). Each bait construct was cotransformed with each prey construct or empty pDEST22 into yeast strain AH109 (Clontech).

To further determine the PIF4 region responsible for the interaction with TCP5, truncated *PIF4* fragments were amplified using primers PIF4-F and PIF4ΔC-R, PIF4ΔN-F and PIF4-R-SC or PIF4-bHLH-F and PIF4-bHLH-R and were then cloned

into pENTR/D-TOPO to generate PIF4ΔC-TOPO, PIF4ΔN-TOPO and PIF4-bHLH-TOPO. The prey constructs PIF4ΔC-AD, PIF4ΔN-AD and PIF4-bHLH-AD were generated via an LR reaction between PIF4ΔC-TOPO, PIF4ΔN-TOPO or PIF4-bHLH and pDEST22. The DBD-TCP5 vector was cotransformed with each prey construct or empty pDEST22 into yeast strain AH109. The bait DBD-TCP5 was cotransformed with each prey construct or empty pDEST22 into yeast strain AH109.

To determine the region in TCP5 responsible for the interaction with PIF4, truncated *TCP5* fragments were amplified using primers TCP5ΔN1-F and TCP5-R, TCP5-F and TCP5ΔTCP-R, TCP5ΔTCP-F and TCP5-R, and TCP5ΔN2-F and TCP5-R and were then cloned into pENTR/D-TOPO to generate TCP5ΔN1-TOPO, TCP5ΔTCP-TOPO and TCP5ΔN2-TOPO. The bait constructs were generated via an LR reaction between pDEST32 and TCP5ΔN1-TOPO, TCP5ΔTCP-TOPO or TCP5ΔN2-TOPO. Each bait construct was cotransformed with PIF4-AD or empty pDEST22 into yeast strain AH109.

The yeast was grown in a 30°C incubator for 3 days. Selection was conducted using SD-Leu-Trp-His (GeneStar) medium with or without 10 mM 3-AT.

Protein extraction and immunoblotting

Plant tissues were ground in liquid nitrogen and then suspended in native buffer (50 mM Tris-MES, pH 8.0, 0.5 M sucrose, 1 mM MgCl2, 10 mM EDTA, 1 mM PMSF) with 5 mM DL-dithiothreitol (DTT) and 1×complete protease inhibitor. The mixtures were kept on ice for 30 min and then centrifuged at 13,000 rpm at 4°C for 15 min. The supernatant was combined with 5×loading buffer (60 mM Tris-HCl, 20% glycerol, 8% SDS, 0.5% Coomassie Brilliant Blue, 2% mercaptoethanol and 10 mM DTT) and then loaded onto 12% SDS/PAGE gels for electrophoresis.

To confirm the interaction between PIF4 and TCPs via co-immunoprecipitation assays, 35Spro-PIF4-Myc and 35Spro-TCPs-Flag were coinfiltrated into tobacco leaves for transient expression as described previously. Total protein from tobacco leaves or transgenic Arabidopsis transformed with both 35S-PIF4-Myc and 35S-TCP5-Flag was extracted and incubated with anti-Flag beads overnight. Anti-Myc or anti-Flag antibodies were used to detect TCP5-Flag or PIF4-Myc. The membranes were incubated in anti-Myc or anti-Actin antibodies diluted 5,000-fold in 3% milk. Anti-Flag antibody conjugated with HRP was diluted 10,000-fold and incubated with the membrane in 3% milk. The secondary goat anti-mouse HRP-conjugated IgG was diluted 5,000-fold. Stable peroxide solution and luminol/enhancer solution (Thermo Scientific, SJ257615) were used for HRP-based detection.

Firefly luciferase complementation imaging

To test the PIF4 interaction with TCPs *in vivo*, PIF4-nLUC was generated via an LR reaction between PIF4-TOPO and pCB1300-nLUC-GW (Zhang et al., 2017). cLUC-

TCPs constructs were generated via an LR reaction between TCPs-TOPO and pCB1300-cLUC-GW (Zhang et al., 2017). PIF4-nLUC and cLUC-TCPs were transformed into Agrobacterium strain GV3101. Different combinations of plasmids were coinfiltrated into tobacco leaves. The plants were grown for 72 hours under long-day conditions. The tobacco leaves were sprayed with 100 mM D-luciferin and kept in the dark for 15 min. The fluorescence was detected using a low-light cooled charge-coupled device (CCD) imaging apparatus (NightOWL II LB983) with indiGO software.

Transient expression analysis in tobacco leaves

The reporter constructs including PRE1pro-LUC, YUC8pro-LUC, PIF4pro-LUC, and PIF4mpro-LUC and the constructs 35Spro-TCP5-Flag and 35Spro-PIF4-Myc were as described above. The control construct, 35Spro-Flag, was generated via an LR reaction between 3Flag-TOPO and pB7FLAGGW2. The constructs were transformed into Agrobacterium strain GV3101. Each reporter was cotransformed into tobacco leaves with pCambia-1300-P19, 35Spro-REN (Guo et al., 2015; Zhang et al., 2017), 35Spro-TCP5-Flag and/or 35Spro-PIF4-Myc, or the control constructs. After incubation for 3 days, the tobacco leaves were treated with luciferin and observed using a CCD imaging apparatus. The firefly (*Photinus pyralis*) and Renilla (*Renilla reniformis*) luciferase activities were measured using dual-luciferase reporter kits with GloMax® 20/20 luminometer (Promega). The fluorescence intensity of REN was used as an internal control. At least three biological repeats were performed.

For dual-luciferase reporter assays of PIF4pro-LUC or PIF4mpro-LUC and TCP5, experimental groups combined with PIF4pro-LUC or PIF4mpro-LUC and 35Spro-3×Flag or PIF4pro-LUC and 35Spro-TCP5-Flag or PIF4mpro-LUC and 35Spro-TCP5-Flag were cotransformed with pCambia-1300-P19 and 35S-REN into tobacco leaves. After 3 days of incubation, the tobacco leaves were treated with luciferin and observed using a CCD imaging apparatus.

Electrophoretic mobility shift assay (EMSA)

An 84-bp probe containing possible TCP binding motifs (5′-GGACCA-3′) in the *PRE1* promoter and its corresponding control probe, in which only the motif (5′-GGACCA-3′) was mutated to 5′-CACCCA-3′, were synthesized and labeled with biotin at the 3′-end by a company (Invitrogen). A 41-bp probe containing possible TCP binding motifs (3′-GGACCA-5′) in the *YUC8* promoter and its corresponding control probe, in which the motif was mutated to 3′-CAGCCA-5′, were synthesized and labeled with biotin at the 3′-end. A 52-bp probe containing possible TCP binding motifs (5′-GGACCA-3′) in the *PIF4* promoter and its corresponding control probe, in which the motif was mutated to 5′-CACCCA-3′, were synthesized and labeled with biotin at the 3′-end. The TCP5-His protein was expressed using a TNT SP6 high-yield wheat germ protein expression system in vitro according to the manufacturer's instructions. The double-stranded

probes were acquired by annealing equimolar concentrations of both complementary oligoes in annealing buffer (10 mM Tris, pH 7.5, 1 mM EDTA, and 50 mM NaCl). EMSAs were performed using a LightShift^{TM} Chemiluminescent EMSA kit according to the manual. Reaction mixtures containing TCP5 protein and probes were incubated at room temperature for 30 minutes, and the products were loaded onto 12% nondenaturing polyacrylamide gels.

RNA-seq analysis

Wild type, *pif4* and *tcp5tcp13tcp17* plants were grown at 20°C for 3 weeks and then transferred to a 28°C growth chamber for 3 days. Total RNA was extracted from the petioles of wild type, *pif4* or *tcp5tcp13tcp17* plants treated with HT for 3 days and from the petioles of the control, which was the wild type continually grown under 20°C. RNA-seq was performed on a HiSeq Illumina Hisequation 2000 platform in the Biomedical Pioneering Innovation Center (BIOPIC) of Peking University. The clean reads were separated from random adapters and low-quality reads. The RNA-seq data were analyzed using a platform from Biomarker Technology Co. (Beijing, China, https://www.biocloud.net/). To find differentially expressed genes (DEGs), HISAT2 (http://ccb.jhu.edu/software/hisat2/index.shtml) was used to align the reads to the TAIR10 and StringTie (https://ccb.jhu.edu/software/stringtie/index.shtml) was used to normalize and calculate the FPKM (fragments per kilobase of exon per million fragments mapped) values as the expression value of each gene. To find the DEGs, RNA-seq data from two samples were analyzed using EBseq (R package version 1.20.0). The false discovery rate (FDR) < 0.01 and fold change ≥ 2 or ≤ -2 were set as the threshold for significantly differential expression. Venn diagrams were made using VENNY (http:// bioinfogp.cnb.csic.es/tools/venny/) with default settings. Heat maps were made using a platform from Biomarker Technology Co. GO analysis were made using DIVID 6.8 (Database for Annotation, Visualization and Integrated Discovery, https://david.ncifcrf.gov/) with default settings.

Chromatin immunoprecipitation PCR (ChIP-PCR)

For ChIP-PCR assays, wild-type control and 35Spro-TCP5-Flag transgenic plants were grown under 20°C for 14 days after germination. Chromatins were sonicated to chip DNA into 200 bp to 500 bp fragments, and then were enriched by Anti-FLAG® M2 Magnetic Beads (Sigma). The DNA fragments was used as templates for qRT-PCR analysis. Relative levels of IP/Input was first normalized by *ACTIN8* (*ACT8*), and then was compared with input DNA samples.

QUANTIFICATION AND STATISTICAL ANALYSIS

Statistical analyses were performed using Microsoft Office Excel 2016. Details of the statistical tests applied, including the statistical methods, number of replicates, mean and error bar details and significances, are indicated in the relevant figure legends. All replicates are biological, unless otherwise noted in the figure legend.
